# Complete mitochondrial genome of an undescribed gnomefish of the genus *Scombrops* (Teleostei, Scombropidae) from southern waters off Kyushu Island, Japan 

**DOI:** 10.1080/23802359.2017.1289348

**Published:** 2017-02-16

**Authors:** Yukako Mochizuki, Riko Yamada, Hirotoshi Shishido, Yasuji Masuda, Shizuko Nakai, Noriyuki Takai, Shiro Itoi, Haruo Sugita

**Affiliations:** a Department of Marine Science and Resources, Nihon University, Fujisawa, Kanagawa, Japan;; b Kagoshima Prefectural Fisheries Technology and Development Center, Ibusuki, Kagoshima, Japan;; c Faculty of Fisheries, Kagoshima University, Kagoshima, Kagoshima, Japan

**Keywords:** Fish, Gnomefish, Scombropid, mtDNA

## Abstract

The complete mitochondrial genome of an undescribed gnomefish species of the genus *Scombrops* was determined using a PCR-based method. The total length of mitochondrial DNA (mtDNA) was 16,521 bp, and included 13 protein-coding genes, two ribosomal RNA (rRNA) genes, 22 transfer RNA (tRNA) genes and one control region. The mitochondrial gene arrangement of this gnomefish species was identical to that of two previously described scombropid species, *Scombrops boops* and *Scombrops gilberti*, and also to those of other teleosts. Maximum likelihood analysis showed that the undescribed scombropid species is most closely related to *S. boops*.

Gnomefish species (genus *Scombrops*) belong to the family Scombropidae, which consists of one genus with three to four species that are found in waters around the Japanese Archipelago (Yasuda et al. 1971; Mochizuki 1979; Itoi et al. 2008, 2010, 2011; Takai et al. 2014), South Africa and Mozambique (Heemstra 1986), and the Caribbean islands (Poey 1860). Recently, we found several individuals of an undescribed scombropid species that inhabits coastal waters off Japan, similarly to the closely related species, *Scombrops boops* and *Scombrops gilberti*. Here, we determined the complete mitochondrial genome sequence of the undescribed scombropid species and investigated the phylogenetic relationship between this species and *S. boops* and *S. gilberti*.

An individual of an undescribed scombropid species with a standard length of 751.3mm and body weight of 8659g was collected from the waters off southern Kyushu Island, Japan, on 11 December 2014. This individual was deposited in the Kanagawa Prefectural Museum of Natural History under catalog number KPM-NI 37952. Total genomic DNA was extracted from muscle, and mitochondrial DNA (mtDNA) sequences were amplified by PCR. Amplification and sequencing of the PCR products were performed following the protocol of Noguchi et al. (2012).

Gene annotation was performed using the online software package MitoFish (http://mitofish.aori.u-tokyo.ac.jp/; Iwasaki et al. 2013); the results of this annotation were manually verified using BLAST searches (https://blast.ncbi.nlm.nih.gov/; Altschul et al. 1997).

The nucleotide sequence of the whole mitochondrial genome (excluding the control region) was aligned using Clustal Omega (Sievers et al. 2011) with those in DDBJ/EMBL/GenBank databases. The alignment was then subjected to phylogenetic inference by means of the maximum likelihood method using MEGA ver. 6.0.6 (Tamura et al. 2013).

A total mtDNA length of 16,521 bp was obtained from the scombropid individual; this sequence included 13 protein-coding genes, two rRNA genes, 22 tRNA genes and one control region (DDBJ accession number LC208773). The heavy (H)-strand contained 29 gene/region sequences comprising 12 protein-coding genes, two rRNA genes, 14 tRNA genes and the control region. The light (L)-strand encoded the remaining genes including one protein-coding gene and eight tRNA genes. Base composition of the mtDNA was 27.6% adenine, 29.5% cytosine, 26.3% thymine and 16.7% guanine. The mtDNA sequence of the undescribed scombropid showed 97.2% and 97.3% identity with those of *S. boops* and *S. gilberti*, respectively (Tsunashima et al. 2016a, 2016b). The mitochondrial gene arrangement was identical to that in *S. boops* and *S. gilberti* and also to those of other teleosts (Miya et al. 2003). Each gene in the mtDNA had the same start and stop codons in the three scombropid species.

A maximum likelihood tree based on mitochondrial genome sequences from 32 teleost species supported the monophyly of Scombropidae, and showed that the undescribed scombropid was most closely related to *S. boops* (Figure 1). Characterization of the mtDNA from the undescribed scombropid will be useful for understanding the relationship among Japanese scombropid species, and for development of molecular tools for investigation of the ecology of these fishes.

**Figure 1.**
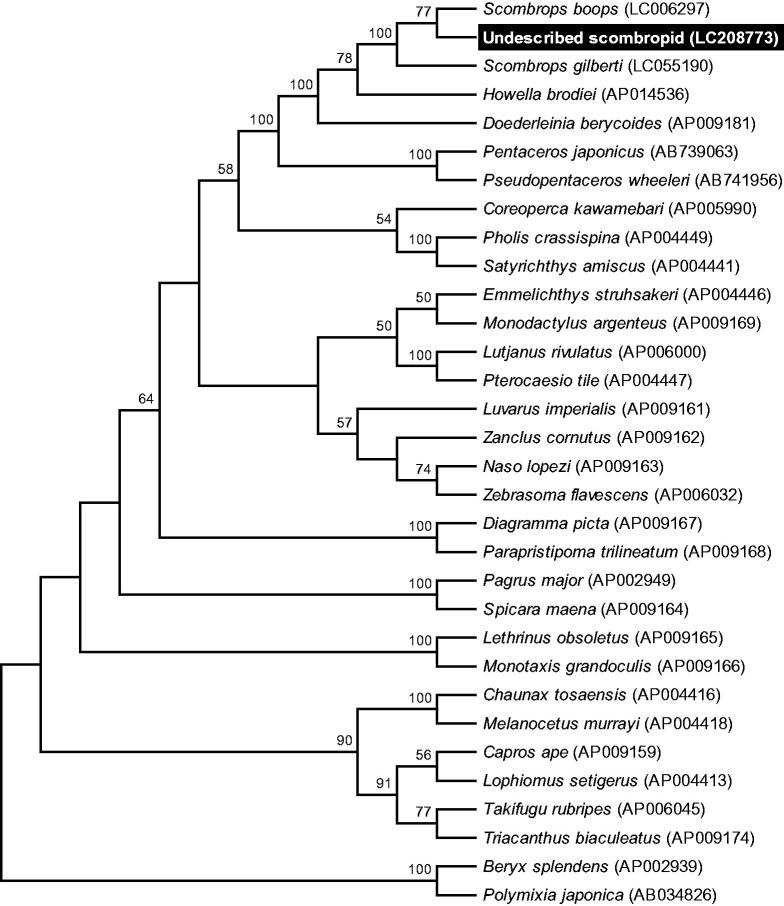
Phylogenetic relationship of the undescribed Japanese gnomefish-related teleost species inferred from the whole mitochondrial genome sequence excluding the control region. The phylogenetic tree was generated by maximum likelihood analysis under the General Time Reversible model. Numbers at branches denote the bootstrap percentages from 1000 replicates. The sequences from *Polymixia japonica* and *Beryx splendens* were used as outgroups. Only bootstrap values exceeding 50% are presented. Data used for the analysis were obtained from *B. splendens* (AP002939), *Capros aper* (AP009159), *Chaunax tosaensis* (AP004416), *Coreoperca Kawamebari* (AP005990), *Diagramma picta* (AP009167), *Doederleinia berycoides* (AP009181), *Emmelichthys struhsakeri* (AP004446), *Howella brodiei* (AP014536), *Lethrinus obsoletus* (AP009165), *Lophius setigerus* (AP004413), *Lutjanus rivulatus* (AP006000), *Luvarys imperialis* (AP009161), *Melanocetus murrayi* (AP004418), *Monodactylus argenteus* (AP009169), *Monotaxis grandoculis* (AP009166), *Naso lopezi* (AP009163), *Pagrus major* (AP002949), *Parapristipoma trilineatum* (AP009168), *Pentaceros japonicas* (AB739063), *Pholis crassispina* (AP004449), *P. japonica* (AB034826), *Pseudopentaceros wheeleri* (AB741956), *Pterocaesio tile* (AP004447), *Satyrichthys amiscus* (AP004441), *Scombrops boops* (LC006297), *Scombrops gilberti* (LC055190), *Spicara maena* (AP009164), *Takifugu rubripes* (AP006045), *Triacanthus biaculeatus* (AP009174), *Zanclus cornutus* (AP009162) and *Zebrasoma flavescens* (AP006032).
